# Ventricular Depolarization Abnormalities and Their Role in Cardiac Risk Stratification — A Narrative Review

**DOI:** 10.31083/RCM25921

**Published:** 2025-01-09

**Authors:** Maarten Blondeel, Tomas Robyns, Rik Willems, Bert Vandenberk

**Affiliations:** ^1^Department of Cardiovasculair Sciences, KU Leuven, 3000 Leuven, Belgium; ^2^Cardiology, UZ Leuven, 3000 Leuven, Belgium

**Keywords:** electrocardiography, arrhythmias, ventricular depolarization, risk stratification, sudden cardiac death, QRS complex, QRS fragmentation, QRS scoring, R-wave heterogeneity, signal-averaged ECG

## Abstract

Ventricular depolarization refers to the electrical activation and subsequent contraction of the ventricles, visible as the QRS complex on a 12-lead electrocardiogram (ECG). A well-organized and efficient depolarization is critical for cardiac function. Abnormalities in ventricular depolarization may indicate various pathologies and can be present in all leads if the condition is general, or in a subgroup of anatomically contiguous leads if the condition is limited to the corresponding anatomic location of the heart. Furthermore, the assessment of depolarization abnormalities on the ECG may either be identified visually or this may depend on further processing. In recent decades, assessment of depolarization abnormalities has received a lot of attention for cardiac risk stratification. This risk stratification aims to identify patients at high risk of adverse cardiac events, to tailor preventive or therapeutic interventions. In this review, we provide an oversight of different techniques for assessing abnormal ventricular depolarization and their value in diagnosing certain conditions, in risk stratification of adverse events, and in guiding therapeutic decisions. This includes QRS alterations directly corresponding to cardiac conditions, such as bundle branch blocks, or the presence of a delta wave, and also metrics aiming to qualitatively or quantitatively assess myocardial scarring, such as QRS (micro)fragmentation and QRS-scoring, and techniques assessing abnormal late depolarizations, such as signal-averaged ECG. While most established assessments of abnormal depolarization rely on human interpretation and are limited by visual detection, recently introduced analyses, such as QRS micro-fragmentation, aim to tackle these limitations. Besides eliminating bias, these automated analyses bypass the need for human interpretation, thereby paving the way for large population studies.

## 1. Introduction

Cardiovascular diseases remain the leading cause of morbidity and mortality worldwide, necessitating effective strategies for prevention and risk stratification [1, 2, 3, 4]. Cardiac risk stratification is a systematic approach to categorize patients based on their potential for adverse cardiac events, such as sudden cardiac death (SCD), myocardial infarction, or heart failure. Effective risk stratification may allow targeted interventions, optimizing patient outcomes by identifying those who would benefit most from specific therapies or preventive measures. While traditional risk factors for cardiac events, such as arterial hypertension, hyperlipidemia, diabetes mellitus, smoking, and family history, are crucial, these factors do not account for all incidences of adverse cardiac events, highlighting the need for more refined risk assessment tools [1].

One of the critical elements in cardiac electrophysiology is ventricular depolarization, which refers to the electrical activation of the ventricles and the subsequent electromechanical coupling to initiate myocardial contraction. Ventricular depolarization is typically assessed using a non-invasive, standard 12-lead electrocardiogram (ECG) where it manifests as the QRS complex [5]. Simple visual assessment of the morphology, duration, and pattern of the QRS complex provides insights into the integrity and functionality of the ventricular conduction system [6]. Abnormalities in ventricular depolarization can arise from various pathological conditions, including myocardial infarction, cardiomyopathies, and electrolyte imbalances [7, 8, 9, 10]. These abnormalities can occasionally be observed in the QRS complex and are often indicative of underlying cardiac pathology [6]. In recent decades, assessment of abnormalities in ventricular depolarization has received significant attention in cardiac risk stratification. Abnormal QRS duration, QRS fragmentation, and the presence of specific conduction blocks are among the markers investigated for their predictive value in identifying high-risk individuals or selecting patients for specific interventions [11].

This narrative review aims to explore the value of ventricular depolarization abnormalities in cardiac risk stratification, supplementing traditional risk factors and enhancing the precision of risk stratification models.

## 2. Ventricular Depolarization on the Surface Electrocardiogram

**Fig. 1.**
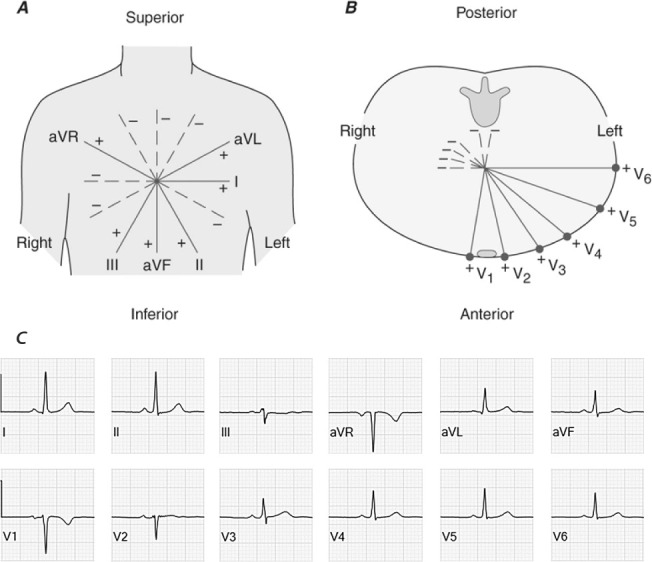
**Schematic representation of the spatial orientation of all 12 leads of the standard surface electrocardiogram**. In the frontal plane (panel A) we distinguish I at 0°, II at 60°, III at 120°, aVL at –30°, aVF at 90° and aVR at –150°. The precordial leads (panel B) comprise V1, positioned in the fourth intercostal space to the right of the sternum; V2 symmetrically to V1 to the left of the sternum; V3 placed diagonally between V2 and V4; V4, 5th intercostal space in the midclavicular line on the left; V5, on the same height as V4 but in the anterior axillary line; and V6, placed on the same level as V4 and V5, but in the midaxillary line. In adults, a normal frontal plane QRS axis is considered to be between –30° and 90°. Panel (C) displays normal electrocardiographic signals for all frontal and precordial leads.

The surface ECG is a cheap indispensable tool in clinical cardiology, providing a non-invasive method to evaluate the electrical activity of the heart (Fig. 1). Using 10 electrodes placed on the patient’s limbs and chest, each of the 12 leads provides an overview of the electrical activity for a specific vector capturing a specific region of the heart in detail. The standard settings for an ECG include a paper speed of 25 millimeters per second (mm/s) and a voltage calibration of 10 millimeters per millivolt (mm/mV), which ensure accurate representation and interpretation of the electrical signals [12]. With digital recordings, the sampling rate has become vital for capturing the precise details of the QRS complex. Higher sampling rates provide better temporal resolution, allowing for more accurate detection and characterization of subtle abnormalities in the QRS complex. Standard ECGs typically operate with a sampling rate between 250 and 1000 Hz, which ensures high-fidelity recording of the rapid electrical activity associated with ventricular depolarization [12]. Holter monitors, which are used for continuous ambulatory monitoring, generally have a lower sampling rate of around 200 Hz. While this is sufficient for most clinical purposes, it may miss finer details detectable at higher sampling rates. Furthermore, proper ECG filtering is also crucial for accurate signal interpretation, including the detection of QRS fragmentation [13]. Filtering of ECG signals may comprise upper- and lower frequency cut-offs, line-frequency rejection and reducing muscle artefacts [14].

## 3. QRS Duration, Morphology, and Amplitude

The typical morphology of the QRS complex includes three main components: the Q-, the R-, and the S-wave [15]. The Q-wave is the initial negative deflection, representing the depolarization of the interventricular septum. Following the Q-wave, the R-wave appears as a positive deflection, indicating the main phase of ventricular depolarization as the electrical impulse spreads through the bulk of the ventricular myocardium. The S-wave is a subsequent negative deflection, reflecting the final phase of ventricular depolarization as the impulse travels towards the base of the ventricles. In general, QRS duration is measured as global interval from the earliest onset to the latest offset of the waveform in all leads [15]. As the QRS duration depends on age and gender, different cut-offs are available. In subjects older than 16 years of age, a QRS duration >110 ms is considered abnormal, while for younger subjects different cut-offs exist depending on the age [15]. A narrow QRS complex suggests that ventricular depolarization is occurring rapidly and efficiently, implying a normal His-Purkinje system. In contrast, a prolonged QRS duration may indicate an abnormal depolarization pattern, often due to conduction delays such as bundle branch blocks or intraventricular conduction delays. In the absence of bundle branch blocks, the difference between maximal and minimal QRS duration across the different leads, a marker known as QRS dispersion, has been associated with adverse outcomes in population studies [16]. The mean frontal plane electrical axis is determined by the maximal QRS deflection, which depends on age and body habitus as it shifts to the left with increasing age. In adults, a normal QRS axis is considered between –30° and 90°. Understanding the nuances of ventricular depolarization on the ECG is essential for accurate diagnosis.

### 3.1 Ventricular Conduction Abnormalities

Abnormal QRS duration and morphology are critical indicators of underlying cardiac dysfunction and can provide essential diagnostic and prognostic information. In cases of specific conduction delays, also called bundle branch blocks, QRS duration and morphology go hand in hand and, although there are some subtle differences between the East and West of the Atlantic, clear diagnostic criteria have been established (Table 1) [11, 15, 17]. Unspecified intraventricular conduction delays comprise all QRS morphologies not fulfilling the criteria for any specific conduction delay but with a QRS duration >110 ms.

**Table 1.** **Criteria to define bundle branch blocks, adapted from Surawicz *et al*. [15]**.

Complete RBBB		
	1	QRS duration greater than or equal to 120 ms in adults.
	2	rsr′, rsR′, or rSR′ in leads V1or V2. The R′ or r′ deflection is usually wider than the initial R wave. In a minority of patients, a wide and often notched R wave pattern may be seen in lead V1 and/or V2.
	3	S wave of greater duration than R wave or greater than 40 ms in leads I and V6 in adults.
	4	Normal R peak time in leads V5 and V6 but greater than 50 ms in lead V1.
Incomplete RBBB		QRS duration between 110 and 120 ms in adults. The other criteria are the same as for a complete RBBB.
Complete LBBB		
	1	QRS duration greater than or equal to 120 ms in adults.
	2	Broad notched or slurred R wave in leads I, aVL, V5, and V6 and an occasional RS pattern in V5 and V6 attributed to displaced transition of QRS complex.
	3	Absent q waves in leads I, V5, and V6, but in the lead aVL, a narrow q wave may be present in the absence of myocardial pathology.
	4	R peak time greater than 60 ms in leads V5 and V6 but normal in leads V1, V2, and V3, when small initial r waves can be discerned in the above leads.
	5	ST and T waves usually opposite in direction to QRS.
	6	Positive T wave in leads with upright QRS may be normal (positive concordance). Depressed ST segment and/or negative T wave in leads with negative QRS (negative concordance) are abnormal.
	7	The appearance of LBBB may change the mean QRS axis in the frontal plane to the right, to the left, or to a superior, in some cases in a rate-dependent manner.
Incomplete LBBB		
	1	QRS duration between 110 and 119 ms in adults.
	2	Presence of left ventricular hypertrophy pattern.
	3	R peak time greater than 60 ms in leads V4, V5, and V6.
	4	Absence of q wave in leads I, V5, and V6.
LAFB		
	1	Frontal plane axis between −45° and −90°.
	2	qR pattern in lead aVL.
	3	R-peak time in lead aVL of 45 ms or more.
	4	QRS duration less than 120 ms.
LPFB		
	1	Frontal plane axis between 90° and 180° in adults.
	2	rS pattern in leads I and aVL.
	3	qR pattern in leads III and aVF.
	4	QRS duration less than 120 ms.

Footnote: RBBB, right bundle branch block; LBBB, left bundle branch block; LAFB, left anterior fascicular block; LPFB, left posterior fascicular block.

The clinical implications of an abnormal QRS duration and/or morphology depend on the presentation. In a diagnostic setting, a left bundle branch block warrants an ischemic work-up, while in a prognostic settings a left bundle branch block may indicate eligibility for cardiac resynchronization therapy in patients with heart failure with reduced ejection fraction [11]. In a randomized trial including patients with left bundle branch block or a bifascicular block which present with syncope, pacemaker implantation reduced adverse events when compared to prolonged rhythm monitoring [18]. Therefore, recognizing these patterns may aid in the selection of appropriate diagnostic and therapeutic interventions, yet this still depends on the clinical presentation. Furthermore, a recent meta-analysis showed an increased risk of all-cause mortality in patients presenting with acute heart failure and a bundle branch block, independent of left or right bundle branch block [19].

### 3.2 Abnormal QRS Voltages

The QRS complex typically has characteristic voltage amplitudes that vary depending on the lead placement and the electrical axis. When these voltages deviate from the normal range, it may indicate underlying pathologies. A low QRS voltage is defined by an abnormally small amplitude of the QRS complex across the standard ECG leads [15]. More specifically, an amplitude less than 5 millimeters in the limb leads or an amplitude less than 10 millimeters in the precordial leads. When observing a low QRS voltage, the following etiologies should be kept in mind: (1) obesity where the excessive body fat attenuates the electrical signals; (2) a pericardial effusion where the fluid accumulation in the pericardium dampens the electrical signals; (3) chronic obstructive pulmonary disease because of hyperinflation of the lungs; (4) cardiomyopathies, such as dilated cardiomyopathy where the intrinsic diseased myocardium generates lower potentials; and (5) infiltrative diseases, such as amyloidosis or sarcoidosis, where the presence of infiltrations disrupt the normal electrical conduction and propagation [20].

Conversely, high QRS voltages may indicate increased myocardial mass or other structural abnormalities. Criteria for high QRS voltage often focus on the amplitude of the R and S waves in specific leads, with common thresholds for the left ventricle including: (1) Sokolow-Lyon Criteria: An R wave in lead V1 plus the S wave in lead V5 or V6 greater than 35 millimeters; and (2) Cornell Voltage Criteria: An S wave in lead V3 plus an R wave in lead aVL greater than 28 millimeters for men and 20 millimeters for women [21]. The right ventricle is more commonly assessed using leads I, V1, and V6 [21]. Frequently associated causes of a high QRS voltage are: (1) Left ventricular hypertrophy, for example, due to longstanding arterial hypertension or aortic stenosis; (2) Right ventricular hypertrophy, for example, due to pulmonary hypertension or congenital heart disease; and (3) athlete’s heart where highly trained athletes present with increased myocardial mass due to extensive remodeling [21].

The clinical implications of an abnormal QRS voltage are mostly diagnostic where the observation of either low or high QRS voltages may trigger further investigations and eventual diagnosis of underlying pathologies. However, the sensitivity and specificity of these abnormalities themselves is rather limited [21].

### 3.3 Other QRS Morphology Abnormalities

**Fig. 2.**
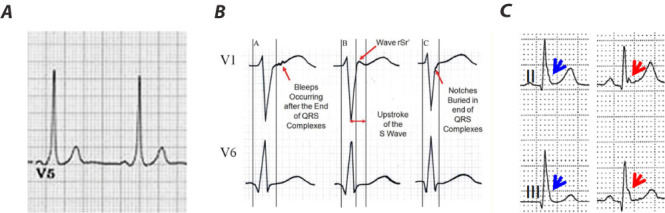
**Examples of delta waves, epsilon waves, and J waves**. Panel (A) displays a delta wave. Note the short PR-interval (<120 ms) and slurred onset of QRS (delta wave). Panel (B) displays different patterns of epsilon waves. The red arrows indicate the 3 patterns of Epsilon waves: (A) wiggle waves, (B) smooth potential waves with QRS duration in V_1_ exceeding the duration in V_6_ by 25 msec. (C) The J wave may be distinct (red arrow) or appear as a slur (blue arrow). In the latter case, part of the J wave is buried inside the QRS. Adapted from Li *et al*. [22] and Antzelevitch *et al*. [23] under the CC BY-NC-ND license (https://creativecommons.org/licenses/by-nc-nd/4.0/).

The QRS complex can exhibit distinct features that have specific clinical implications. First, delta waves are pathognomonic for pre-excitation syndromes, most often due to atrioventricular accessory pathways. The diagnosis of a delta wave refers to a typical slow upstroke of the QRS associated with a short PR interval (<120 ms in adults) and a widening of the QRS complex (Fig. 2A, Ref. [22, 23]), often accompanied by abnormal repolarization due to T wave memory. In general, accessory pathways are often present early during fetal development and spontaneously disappear. Furthermore, approximately 50% of accessory pathways degenerate before 1 year of age [24, 25]. Preexcitation syndrome generally is benign, either asymptomatic or revealed by paroxysmal tachycardia, such as in the Wolff-Parkinson-White syndrome. SCD in preexcitation syndrome is related to rapid conduction of atrial fibrillation to the ventricles with degeneration into ventricular fibrillation. SCD incidence however is low and rare in children with an incidence of 0.02% per year, while the incidence of supraventricular tachycardia is approximately 1% per year. High risk features of an accessory pathway include younger age, documented atrioventricular reentrant tachycardia (AVRT), evidence of multiple accessory pathways, and the conduction properties of the accessory pathway. This includes an antegrade effective refractory period or shortest pre-excited RR interval during atrial fibrillation ≤250 ms [26].

Epsilon waves, firstly described in 1997 by Fontaine, are low-amplitude, positive deflections at the end of the QRS complex (Fig. 2B), typically in the right precordial leads, and are a well-described feature in arrhythmogenic right ventricular cardiomyopathy (ARVC) [22, 27]. The prevalence of epsilon waves varies between 0.9% and 2.5% [28]. In ARVC myocytes in the right ventricle are progressively replaced by fat resulting in islands of excitable myocytes. Epsilon waves are believed to be the result of the late excitation of myocytes in these islands surrounded by fat tissue. While they are quite specific for ARVC and are among the minor diagnostic criteria defined in the 2024 expert consensus statement, they have also been observed in patients with a posterior myocardial infarction, right ventricular infarction, and infiltrative diseases [28, 29].

While epsilon waves are an electrical sign of late depolarization, several repolarization markers at the level of the J point have also been described. In general, J waves refer to a rare, slow deflection of uncertain origin. Osborn waves, a type of J wave (Fig. 2C), are dome-shaped deflections most commonly observed in the inferior and lateral leads in patients presenting with hypothermia [30]. However, Osborn waves are not pathognonomic for hypothermia and in fact, non-hypothermic Osborn waves comprise a long list of distinct causes, including hypercalcemia, acute myocardial ischemia, Takotsubo cardiomyopathy, left ventricular hypertrophy, and Brugada syndrome [31, 32, 33, 34]. While these are typically considered benign features, they have been associated with an increased risk of SCD [35]. While there is strong evidence that the underlying electrophysiological mechanisms of J-waves are related to an injury current associated with repolarization, the debate about whether delayed depolarization is involved remains present [30].

## 4. Signal-Averaged ECG

**Fig. 3.**
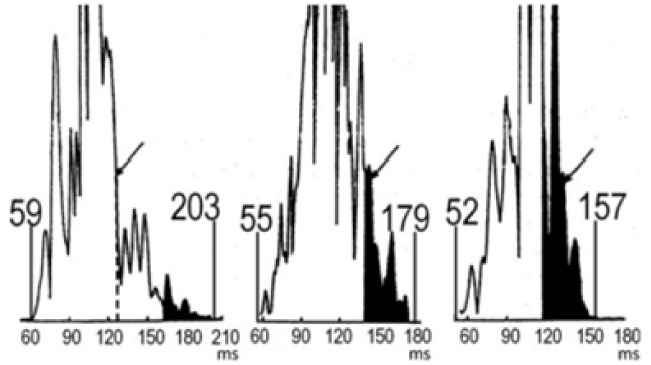
**Example of signal-averaged ECGs**. Signal averaged ECGs for, from left to right, a patient with Naxos Syndrome (comprising arrhythmogenic right ventricular dysplasia), a post-myocardial infarction patient with sustained VT, and a healthy control. Adapted from Gatzoulis *et al*. [38] under the CC BY-NC-ND license (https://creativecommons.org/licenses/by-nc-nd/4.0/). ECGs, electrocardiograms; VT, ventricular tachycardia.

The signal-averaged ECG originates from the early 1970’s in an effort to non-invasively detect the electrical activity of the His bundle [36]. Soon thereafter, the aim transitioned towards the detection of late ventricular potentials which are believed to reflect the depolarization of ventricular areas with slow conduction, hence a substrate for ventricular arrhythmias. As these subtle signals are often masked by noise, a signal-averaged ECG averages multiple high-resolution ECG signals (≥1000 Hz) to reduce noise [37]. Late ventricular potentials (Fig. 3, Ref. [38]) are low-amplitude, high-frequency signals in the terminal part of the QRS complex and are defined using 3 criteria: (1) the duration of the filtered QRS complex ≥114 ms; (2) the root mean square voltage of the terminal 40 ms of the QRS complex (RMS-40) ≤20 microVolts (µV); and (3) the duration of low amplitude signals less the 40 µV (LAS-40) >38 ms. A second signal of interest, although it never reached extensive clinical testing, are intra-QRS potentials [39]. These are low amplitude notches in the QRS complex not visible in the routine 12-lead ECG, but may be observed in the signal-averaged QRS complex without prolonging the QRS duration. In retrospect, the attempts to extract intra-QRS potentials may be considered the first efforts to automatically detect fragmented QRS complexes.

The interest in the signal-averaged ECG was based on the observation that in over 90% of patients presenting with sustained monomorphic ventricular tachycardia after myocardial infarction, late ventricular potentials were observed, while this was 20% or less in the absence of ventricular arrhythmias [40]. Furthermore, the signal-averaged ECG yielded a high negative predictive value with normal recordings almost excluding a substrate for reentrant ventricular tachycardia [41]. The potential of the signal-averaged ECG was subsequently investigated in the MUSTT trial (Multicenter Unsustained Tachycardia Trial) where in specific subgroups the measures of the signal-averaged ECG were predictive of arrhythmic events as well as cardiac mortality [42]. Despite these promising results, the positive predictive value of the signal-averaged ECG was rather low resulting in a decline in its use. Until recently, late potentials on signal-averaged ECG were a minor diagnostic criterium for ARVC. In the proposed diagnostic criteria of 2024, however, it is no longer considered due to low diagnostic accuracy [28, 29].

## 5. QRS Fragmentation

### 5.1 Macro-Fragmentation

**Fig. 4.**
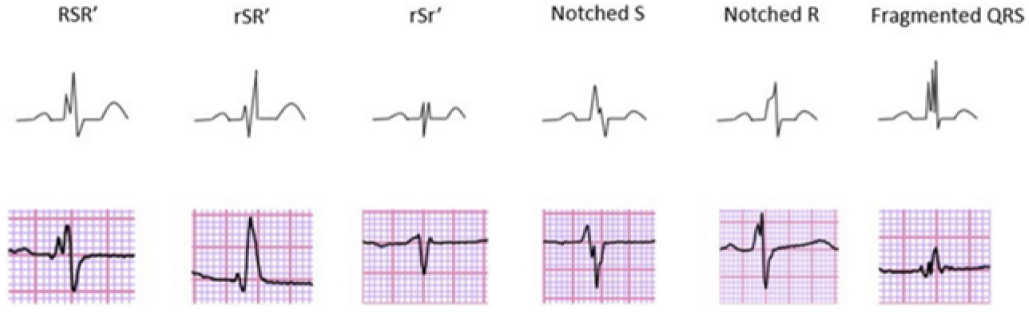
**Different morphologies of fragmented QRS on 12-lead ECG describing the fQRS criteria proposed by Das and Zipes [43]**. Adapted from Haukilahti *et al*. [44] under the CC-BY license (https://creativecommons.org/licenses/by/4.0/). ECG, electrocardiogram; fQRS, fragmented QRS.

Fragmented QRS (fQRS) or QRS macro-fragmentation (Fig. 4, Ref. [43, 44]) was first described in 1969 by Flowers *et al*. [45]. It is characterized by notching or slurring in the QRS signal and serves as a qualitative marker of inhomogeneous ventricular depolarization mainly due to aberrations in the myocardium, primarily hypothesized to be fibrosis or scarring [46, 47, 48]. fQRS has been repeatedly associated with adverse outcomes in cardiac patients [49, 50]. Following the criteria defined by Das and Zipes [43] in cases of a normal QRS duration, fQRS is usually defined as the presence of various RSR’-patterns in 2 or more anatomically contiguous leads, i.e., inferior, anterior, or lateral, in a standard 12-lead ECG [43, 49, 50, 51]. In cases of a wide QRS complex, the presence of >2 notches on the R wave or the S wave in ≥2 contiguous leads is required [52]. The physiological mechanism of the electrocardiographic findings has been explained by shifting of the QRS vector in and around myocardial scar or fibrosis during ventricular depolarization [43]. Despite numerous attempts, fQRS could not be directly correlated with myocardial scar or fibrosis. A large Finnish study of >12,000 patients suggested that fQRS is present in +/– 20% of patients without known heart disease [50]. In a retrospective analysis of the MADIT-II trial, fQRS was only present in around one third of patients, while all patients had extensive ischemic heart disease with a left ventricular ejection fraction ≤30% [53]. Additionally, there was no association with the anatomic location of fQRS and Q-waves [53]. Lastly, Morita *et al*. [54] showed, using an isolated canine right ventricular tissue model with pharmaceutically induced Brugada syndrome (or rather Nava-Martini-Thiene syndrome), that activation delay in the epicardium could reproduce similar fQRS patterns [54, 55]. These findings suggest that fQRS is not solely a representation of myocardial scar or fibrosis.

A meta-analysis conducted by Rosengarten *et al*. [49] showed a significant association between fQRS and both all-cause mortality and SCD in patients with ischemic or non-ischemic heart disease. Interestingly, fQRS was not significantly associated with increased mortality in the subgroup of patients with a cardiac implantable electronic device, predominantly implantable cardioverter-defibrillators (ICD), whereas it was associated with mortality in the whole population [49]. Since patients carrying an ICD are largely protected against SCD, this suggests that fQRS may be more suited as a marker of SCD risk rather than all-cause mortality [49]. Another meta-analysis by Engstrom *et al*. [46] including patients with heart failure and left ventricular ejection fraction ≤40% also found a significant association between fQRS and both ventricular arrhythmias (VA) and all-cause mortality. Terho *et al*. [50] conducted a large study of approximately 11,000 Finnish middle-aged subjects and found a significant association between presence of fQRS and cardiac mortality in patients with cardiac disease, but not with all-cause or arrhythmic mortality. Interestingly, in subjects without known heart disease, they found no significant association with any mortality-related outcome [50]. They proceeded to analyze whether different anatomic locations of fQRS had different prognostic implications. In these subgroup analyses, only lateral fQRS was significantly associated with arrhythmic mortality [50]. In this context, Vandenberk *et al*. [56] conducted a study of 400 patients with an ICD in primary prevention. They found that fQRS in inferior leads was associated with VA, whereas anterior fQRS was rather related to all-cause mortality despite ICD-protection [56]. A meta-analysis regarding cardiac resynchronization therapy (CRT) showed that the presence of fQRS was independently associated with non-response to CRT [57]. CRT response was defined as <15% decrease in left ventricular end-systolic volume at follow-up in 3 out of 7 included studies, but was not defined in 4 of the included studies. Since predicting CRT response prior to implantation remains challenging, they suggested that the absence of fQRS could be of value in selecting ideal CRT candidates.

fQRS has also been associated with adverse outcomes in Brugada patients and in patients with ARVC. In Brugada syndrome, primarily considered a channelopathy, fQRS is seen more frequently in carriers of a mutation in *SCN5A*-gene, and it is associated with the occurrence of ventricular fibrillation (VF) and syncope, possibly guiding ICD implantation in these patients [54, 58]. A meta-analysis in ARVC patients showed that fQRS was significantly associated with mortality, appropriate ICD shocks, and SCD [59].

### 5.2 QRS Micro-Fragmentation

The main limitation of QRS macro-fragmentation is the visual interpretation, often in retrospective studies, resulting in a binary, non-quantifiable classification prone to inter- and intra-observer variability [60]. Since the early 1990s, efforts have been made to quantify abnormalities and turbulence in the spatial distribution of intra-ventricular conduction below the resolution of visual detection, based on spectral analyses of signal-averaged ECGs [61, 62]. More recently, Hnatkova *et al*. [63] introduced the concept of QRS micro-fragmentation (QRS-µf) as a new method for quantifying microscopic depolarization abnormalities. It is important to note that QRS-µf should not be interpreted as a refined version of macro-fragmentation, but that it rather detects signal characteristics invisible to the naked eye and is therefore independent of fQRS [63].

**Fig. 5.**
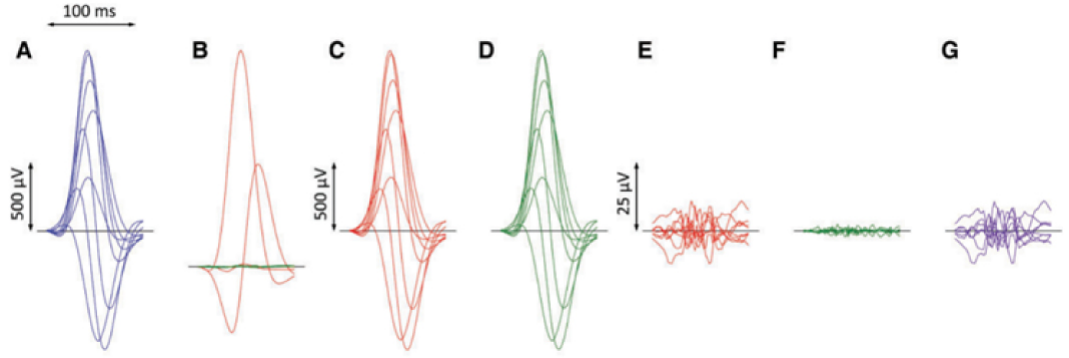
**ECG processing for calculation of QRS micro-fragmentation**. Filtered QRS complex patterns of independent Leads I, II, V1, V2, …, V6 are considered together as if on the same isoelectric axis (A). Singular value decomposition transforms the signals into eight algebraically orthogonal signals which are sorted according to their contribution to the original ECG leads (components 1–3 are shown in red, 4–6 in green, and 7 and eight in amber in panels (B); the 7th and 8th components are almost invisible in these cases). The components 1–3 create the optimized three-dimensional QRS vector projection. When these components are used to reconstruct the original ECG, patterns in panels (C) are obtained while reconstruction based on components 1–6 gives patterns in panels (D). (E,F) show the differences between the original ECG are the reconstruction based on 1–3 and 1–6 components, respectively (i.e., E = A–C, F = A–D). The residuals shown in panels (F) (corresponding to the contribution of 7th and 8th components) are considered noise and eliminated. QRS micro-fractionation is calculated as the averaged absolute area under contribution by components 4–6 shown in panels (G) (G = D–C = E–F). Reproduced from Hnatkova *et al*. [63] under the CC BY license (https://creativecommons.org/licenses/by/4.0/). ECG, electrocardiogram.

QRS-µf is calculated as the difference between an original ECG and the reconstructed ECG after projecting the original ECG into optimized three perpendicular dimensions (Fig. 5) [63, 64]. Put simply, a 12-lead ECG can be reduced to 8 algebraically independent leads (leads I, II, and precordial leads V1–V6) without losing information [63, 64]. Applying singular value decomposition (SVD), these 8 leads can be spatially rotated and rescaled into 8 optimized orthogonal components, representing a theoretical 8-dimensional space, sorted according to their contribution to the original ECG [63, 64]. The first component captures the direction with the largest QRS signal, the second component is perpendicular to the first in the direction where it again maximally contains the QRS signal, and so on, always taking as much of the signal as possible into the next dimension [63, 64]. Consequently, the first three components correspond to a 3-dimensional representation of the ECG, while the remaining components 4 to 8 go beyond 3 dimensions. As a next step, an 8-lead ECG is reconstructed out of components 1, 2 and 3. QRS-µf is calculated as the difference in total area under the absolute QRS complex curves between the original ECG and the reconstructed ECG out of components 1 to 3, minus components 7 and 8 since these are considered noise. This equals the contribution of components 4 to 6 cumulatively. Based on observations in healthy subjects, it is proposed that QRS-µf of >3.5% might be considered abnormal.

Hnatkova *et al*. [63] proceeded to analyze QRS-µf as a predictor of adverse outcomes in three populations of different clinical characteristics, specifically the population of (1) the EU-CERT-ICD study [65], comprising recipients of an ICD in primary prevention, (2) the VA Washington study [66], comprising US male veterans with ischemic and non-ischemic heart disease, and (3) the Whitehall II study [67], comprising British civil servants with sinus rhythm. In all analyzed populations increased QRS-µf was strongly associated with all-cause mortality, independent of other established risk factors and fQRS [63]. Since all-cause mortality was a reasonable approximation of cardiovascular mortality in the first 2 populations, and since there was no association between QRS-µf and non-cardiovascular mortality in the third population, QRS-µf seems to be associated with cardiovascular mortality. Since the strongest association with all-cause mortality was noted in the EU-CERT-ICD population, which comprises patients protected by an ICD, it is likely that QRS-µf rather predicts death related to heart failure instead of VA. This is further supported by QRS-µf being only borderline significantly predictive of a first appropriate ICD-shock in a univariate analysis [63]. Importantly though, anti-tachypacing interventions were not included in this analysis, possibly concealing value in predicting VA [63]. Interestingly, a strong association with mortality was also noted in a sub-population from the EU-CERT-ICD population with atrial fibrillation [63]. This is of importance since previously defined ECG-based risk stratifiers are not applicable in atrial fibrillation [63]. Moreover, fQRS lost significance when including QRS-µf in a multivariate analysis, or when excluding patients with QRS-µf >3.5%, while QRS-µf kept predictive value when excluding patients with fQRS present, suggesting that QRS-µf is a more potent predictor of mortality than fQRS [63].

## 6. QRS Non-Planarity

**Fig. 6.**
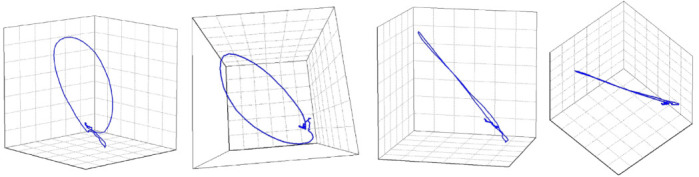
**A visual representation of a QRS-planarity assessment**. The image shows that the loop of the QRS complex was planar, that is, that it collapses into a practically straight line when viewed from the side of the plane of the QRS vector movement. The QRS non-planarity (i.e., the departures from the plane of the vector movement) was 2.17%. Adapted from Hnatkova *et al*. [68] under the CC BY license (https://creativecommons.org/licenses/by/4.0/).

It has already been established that in healthy hearts the vectorcardiography (VCG) loops of the QRS-complex are almost entirely planar (Fig. 6, Ref. [68]), and that in unhealthy hearts, for example after myocardial infarction, the VCG loops tend to twist out of plane especially when the disease is extensive [69, 70, 71, 72]. Within this context, Hnatkova *et al*. [68] tested the hypothesis that QRS non-planarity, as another marker of abnormal depolarization, could predict adverse outcomes in cardiac patients. Applying the same technique as described earlier, component 3 captures the electrical signals that go out of one plane (since planar signals are captured by components 1 and 2), and are therefore labelled QRS-np [68]. Although strictly not in the scope of this review, T-wave non-planarity can be calculated in a similar manner. Hnatkova *et al*. [68] conducted a retrospective analysis on the EU-CERT-ICD population, comprising patients with an ICD in primary prevention. QRS-np was independently associated with mortality despite ICD-protection [68], similarly to QRS-µf, indicating that these abnormalities in depolarization are mainly associated with poorer survival in cardiac patients being caused by heart failure progression rather than VA [43, 63]. Interestingly, T-wave non-planarity was independently associated with appropriate ICD shocks [68], consistent with the repeated association of repolarization abnormalities and VA and SCD [43, 73, 74].

## 7. R-Wave Heterogeneity

ECG heterogeneity is defined as nonuniformity in depolarization or repolarization, which leads to heterogeneity in R-wave or T-wave morphology, respectively [75]. Spatial heterogeneity calculation is based on second central moment analysis and it measures simultaneous dissimilarities across typically 3–5 ECG leads [75]. Firstly, a mean morphology for the QRS or T-wave is generated out of a standard surface ECG. R-wave or T-wave heterogeneity (TWH) is then calculated by taking the square root of the variance about the mean morphology [76, 77, 78]. Since this review focuses on depolarization abnormalities, we will specifically concentrate on R-wave hteterogeneity (RWH). In patients hospitalized with decompensated heart failure, Nearing *et al*. [79] showed that RWH (and also TWH) in leads V1, V5 and aVF elevated in the last 30–45 minutes before the onset of VA. Notably, an increase in RWH and TWH preceded the appearance of T-wave alternans (TWA), a marker of repolarization instability strongly associated with short term VA, by 15 minutes [79]. Consequently, RWH and TWH could potentially provide an earlier warning for impending VA in patients hospitalized for heart failure. One could argue that this short interval before arrhythmia onset is suboptimal for clinical implementation, but RWH was also validated with longer follow-up periods. Kenttä *et al*. [76] proceeded to analyze RWH, TWH or J-wave heterogeneity (JWH) in the left precordial leads (V4–V6) in a large cohort of Finnish adults without prior conduction abnormalities, to assess whether depolarization or repolarization heterogeneity could predict SCD in the general population. With a mean follow-up period of almost 8 years, RWH was found to be predictive of SCD, albeit only univariately [76]. When RWH was present with simultaneous present JWH and TWH, the univariate hazard ratio for SCD rose to 9.0 (95% CI 4.2–19.3, *p* < 0.001), with significant predictive value persisting in a multivariate analysis [76]. These findings suggest that RWH, JWH and TWH in the lateral precordial leads could prove useful as a readily available, non-invasive screening tool for SCD in the general population [76]. In another study by Tan *et al*. [80], RWH was assessed in 120 patients who presented for an electrophysiologic study, ventricular tachycardia (VT) ablation, ICD implantation, or generator change, without apparent reversible trigger for VA. With a mean follow-up period of 31 months, abnormal RWH and TWH identified 67% of patients who would have malignant VAs or appropriate ICD therapy, and 85% of patients who would proceed to develop SCD, either fatal or resuscitated [80]. In patients with present cardiomyopathy, a significantly diminished arrhythmia-free survival when RWH and/or TWH was present, independent of age, sex and left ventricular ejection fraction [80]. Besides arrhythmias, RWH has also been associated with CRT non-response. Bortolotto *et al*. [81] showed that in 35 CRT-recipients without left bundle branch block, pre-implantation RWH was significantly lower in mechanical super-responders (defined as ≥20% increase in left ventricular ejection fraction and/or ≥20% decrease in left ventricular end-systolic diameter) with an area under the curve reaching up to 0.891, while pre-implantation QRS-duration was not significantly predictive.

## 8. QRS Scoring

Starting halfway the 20th century, there was a growing interest in “imaging” the heart, or more specifically scar tissue in the heart, through ECG and VCG signals. Selvester *et al*. [82] showed, starting in the 1960s, that scars in different parts of the ventricles produced characteristic and quantifiable changes in the ECG and VCG, and developed scores that considered Q- and R-wave durations, R/Q and R/S amplitude ratios, R- and S-wave amplitudes and R-wave notches [82, 83, 84, 85, 86]. These scores were mainly created to determine infarction location and size, and were extensively validated through correlation with post-mortem studies and, later, cardiac magnetic resonance imaging with late gadolinium enhancement [83, 84]. In 2008, a revised version of the Selvester score was also shown to identify and quantify scarring in non-ischemic cardiomyopathies with left ventricular ejection fraction ≤35%, even in the presence of confounders like hypertrophy, or bundle branch or fascicular blocks [84]. As to being related to outcome, Strauss *et al*. [87] tested QRS scoring in the population of the ICD arm of the Sudden Cardiac Death in Heart Failure Trial (SCD-HeFT), and found that increasing QRS score corresponded with increasing infarction size and predicted higher rates of VA. Patients with a score of 0 had 48% fewer VA [87]. Combining QRS score (absence vs. presence) with left ventricular ejection fraction distinguished risk groups with 73% fewer VA in low risk group compared to the high risk group [87]. Furthermore, retrospectively studying an automated version of the Selvester score in the EU-CERT-ICD population, comprising ICD recipients in primary prevention, Reichlin *et al*. [88] showed that a score of ≥5 was independently associated with increased mortality. In a study by Sweeney *et al*. [89], higher QRS scores were associated with reduced reverse remodeling in patients with CRT with left ventricular ejection fraction ≤35%.

## 9. Future Perspectives

The discussed analyses of abnormal ventricular depolarization provide valuable insights and have demonstrated significant associations with adverse outcomes in both patients with cardiovascular diseases and in the general population. However, techniques like fQRS or QRS scoring depend on visual interpretation, resulting in high intra- and interobserver variability, and consequently introduce bias. Furthermore, these techniques are limited by the resolution of the human eye, thereby only detecting macroscopic alterations in the QRS complex. Therefore, future endeavors should focus on automated techniques and metrics that quantify alterations invisible to the naked eye, also called “invisible electrocardiography” [90]. Recently, QRS-µf and QRS-np have emerged as novel quantitative methods that overcome these limitations of traditional visual detection. In the first studies, these analyses have shown promise in predicting heart failure progression, as well as, together with T-wave non-planarity, differentiating non-arrhythmic mortality from VA [68]. Since risk stratification for VA remains very challenging, these analyses may prove to be of value in identifying ideal candidates for ICD implantation. Further studies are needed, but these findings could have a significant impact on the prediction of which patients would benefit from ICD implantation. Since the incidence of SCD has decreased in the last decades, current risk stratification methods are a matter of debate [91]. If the predictive value of QRS-np and T-wave non-planarity can be validated in independent cohorts, demonstrating their ability to differentiate between the competing risks of appropriate ICD therapy and ICD resistant mortality, these ECG-based parameters hold the potential to revolutionize risk stratification of VA and SCD, and consequently change current clinical practice in identifying ideal candidates for ICD implantation. Another major challenge is conducting high-quality studies. Usually, studies assessing ECG metrics are of a retrospective nature, which is an important source of bias, especially when the investigated metrics rely on visual interpretation. Automated techniques eliminate bias related to intra- and interobserver variability. Additionally automated analyses allow a swift analysis of hundreds of thousands of ECGs, instead of being limited by human interpretation, thereby paving the way for large population studies, for example using contemporary single- or multi-lead wearable device recordings.

## 10. Conclusions

This review summarizes established methods of assessing ventricular depolarization abnormalities. These abnormalities can manifest macroscopically on a routine 12-lead ECG or at the microscopic level requiring post-processing. Metrics such as fQRS and QRS scoring aim to assess the presence of scar tissue in the myocardium, qualitatively and quantitively respectively, while signal-averaged ECG’s assess late depolarizations. Scar tissue and late depolarization both serve as a potential arrhythmic substrate. So not surprisingly, these metrics have been repeatedly associated with VA and SCD. Future endeavors should focus on automated techniques and metrics that quantify these alterations invisible to the naked eye. Recently introduced analyses such as QRS-µf and QRS-np hold significant potential, but require large population studies to establish their role in future risk stratification.
